# E3 Ubiquitin Ligase SPL2 Is a Lanthanide-Binding Protein

**DOI:** 10.3390/ijms22115712

**Published:** 2021-05-27

**Authors:** Michał Tracz, Ireneusz Górniak, Andrzej Szczepaniak, Wojciech Białek

**Affiliations:** 1 Department of Biophysics, Faculty of Biotechnology, University of Wrocław, Joliot-Curie 14a, 50-383 Wrocław, Poland; michal.tracz@uwr.edu.pl (M.T.); ipg3kg@virginia.edu (I.G.); andrzej.szczepaniak@uwr.edu.pl (A.S.); 2 Department of Molecular Physiology and Biological Physics, University of Virginia School of Medicine, Charlottesville, VA 22908, USA

**Keywords:** ubiquitination, chloroplast, lanthanides, SPL2

## Abstract

The SPL2 protein is an E3 ubiquitin ligase of unknown function. It is one of only three types of E3 ligases found in the outer membrane of plant chloroplasts. In this study, we show that the cytosolic fragment of SPL2 binds lanthanide ions, as evidenced by fluorescence measurements and circular dichroism spectroscopy. We also report that SPL2 undergoes conformational changes upon binding of both Ca^2+^ and La^3+^, as evidenced by its partial unfolding. However, these structural rearrangements do not interfere with SPL2 enzymatic activity, as the protein retains its ability to auto-ubiquitinate in vitro. The possible applications of lanthanide-based probes to identify protein interactions in vivo are also discussed. Taken together, the results of this study reveal that the SPL2 protein contains a lanthanide-binding site, showing for the first time that at least some E3 ubiquitin ligases are also capable of binding lanthanide ions.

## 1. Introduction

Lanthanide probes and lanthanide-binding tags (LBT) present attractive photophysical and magnetic properties for cellular biologists and biophysicists. Their significant advantages over conventional fluorophores are not limited to their unique emission profiles or extremely long-lived luminescence [1] but extend to their resistance to photobleaching and the specificity of the labeling. In addition, the small size of LBTs is of paramount importance as it results in a low probability of adverse effects exerted on protein functions. These features make LBTs particularly well-suited for luminescence and imaging and explain why Ln-based probes have gained much interest in recent years. Some of the latest applications involve phasing protein X-ray crystal structures using only the anomalous signal [2] or in vivo visualization of specific proteins tagged with X-ray-sensitive peptide sequences to improve the image resolution beyond the diffraction limit of visible light [3].

It had long been known that lanthanides could replace Ca^2+^ in biological systems, but it was not until 2011 that a specific biological role of lanthanides was demonstrated. XoxF, a naturally occurring lanthanide-binding protein, was identified from *Methylobacterium extorquens* and reported to increase methanol dehydrogenase activity [2]. Another natural, and probably the best-studied, rare earth element (REE)-binding protein is lanmodulin (LanM). Since some REEs are now widely used in high-technology industries, LanM offers an attractive possibility for extracting rare earth metals, especially since the protein offers unprecedented selectivity against non-REE elements under acidic pH and high temperature while outperforming many chemical processes currently in use [3]. Consequently, lanthanides are now regarded as essential cofactors in certain enzymes of methylotrophic bacteria. Apart from the technological application of Ln-binding enzymes, several reports have been published supporting the view that small amounts of REEs may favor biomass production of vascular plants (reviewed in [4]). Therefore, REEs are now commonly found in fertilizers across China to improve crop production [5].

The cytosolic ubiquitin–proteasome system (UPS) controls the quality of eukaryotic proteins. Ubiquitin activating (E1), ubiquitin conjugating (E2), and ubiquitin ligating (E3) enzymes mediate the attachment of ubiquitin to target proteins. Available results not only show that this posttranslational modification of substrate proteins can result in their proteasomal degradation, modulation of localization, protein–protein interactions or modification of activities, but they also implicate the ubiquitin pathway in signal transduction, the immune response, DNA damage repair, endocytosis, and cell-cycle progression [6]. This unequivocally highlights the importance of ubiquitination in nearly all aspects of eukaryotic biology.

In view of the diversity of ubiquitination substrates and their cellular roles, selecting appropriate proteins for ubiquitination is critical. Substrates for ubiquitination are identified primarily by the E3 ligases, both the RING (Really Interesting New Gene) or HECT (Homologous to the E6-AP C-terminus)-type enzymes. Recently, SP1, and its two homologs, SPL1 and SPL2, have been identified as RING-type ubiquitin E3 ligases found in *Arabidopsis thaliana* [7], the model organism for research in plant biology. Importantly, these were the first E3 ligases identified in chloroplasts, specifically in their outer envelope membrane, thus indicating that the chloroplast proteome, similarly to the mitochondrial one, is also strictly controlled by the UPS. While SP1 was shown to ubiquitinate TOC components that control protein import from the cytosolic compartment to chloroplasts [7,8], the function of both SPL1 and SPL2 (AGI loci: AT1G54150) remains obscure. In the case of all three identified chloroplast E3 ligases, their RING domains, responsible for their catalytic functions, are exposed to the cytosol. Among these homologous proteins, the amino acid sequence of SPL2 is the longest due to several insertions. Interestingly, one of them resembles sequences of lanthanide-binding tags (LBT). Therefore, we have decided to investigate the putative binding of lanthanides to SPL2, mainly by means of circular dichroism (CD) and fluorescence spectroscopy. To the best of our knowledge, this is the first report of E3 ubiquitin ligase-binding lanthanide ions.

## 2. Results

### 2.1. Sequence Analysis and Prediction of Lanthanide-Binding Motif

**Figure 1:**
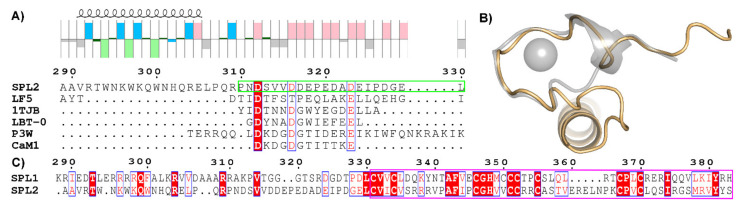
Comparison of sequences and structures of SPL2 and lanthanide-binding peptides. (**A**) Sequence alignment of the part of SPL2 showing its cytosolic region without RING, LF5—lanthanide finger 5 [11], 1TJB—high-affinity LBT peptide [10], LBT-O—peptide optimized for terbium luminescence [12], and P3W—DNA-dependent MRI contrast agent [12]. For comparison, one of the Ca2+-binding sites of calmodulin is shown (CaM1). The predicted disordered region is outlined in light green. A hydropathy plot of SPL2 revealing the position of basic (blue), acidic (pink), aliphatic (gray), polar (dark green), and aromatic (light green) amino acid residues. Predicted secondary structures of SPL2 are depicted on top. The predicted structure of the MBP-SPL2cyt fusion protein is shown in Figure A1 (Appendix A). (**B**) Structural alignment of the LBT–Tb3+ complex (1TJB, gray) and the predicted cytosolic region of SPL2 (gold). Terbium ions bound to 1TJB are shown as a gray sphere. (**C**) Amino acid sequence alignment of the cytosolic domain of the SPL1 and SPL2 proteins. RING domains are boxed in pink.

SPL2 is a 383-amino-acid-long protein localized to the outer membrane of chloroplasts. Two membrane-spanning regions (residues 14–34 and 268–288) are predicted to separate its intermembrane-space domain (35–267) from cytosol-exposed N-(1–13) and C-termini (289–383). The latter comprises RING (residues 331–383) and the 42-residue-long linker region (289–330) that separates transmembrane and RING domains. Strikingly, the linker region is mainly composed of disorder-promoting residues, as defined by Campbell et al. [9]; thus, we predict it to be intrinsically disordered. Intriguingly, a part of the linker shares partial sequence similarity with lanthanide-binding peptides, as suggested by multiple sequence alignments (Figure 1A). This analysis also indicated that SPL2 affinity for Ln ions might not be as high as in the case of the iteratively designed and tested LBTs shown in Figure 1.

To gain further insight into the binding of Ln ions by SPL2, we performed structural modeling of its linker region based on the known structure of the Tb-binding LBT peptide (1TJB) [10]. The overall fold of the predicted Ln-binding motif is similar; however, there are significant differences at the sequence level (Figure 1). In the case of this chemically evolved LBT, its sequence was carefully designed to achieve a nM-level affinity for Ln ions. This is accomplished by oxygen ligands of D1, N3, and D5, carboxylate ligands from E9 and E12, and the backbone carbonyl group of W7 that form the eight-coordinate Tb^3+^ complex [7]. Since not all these residues are present at the corresponding positions in SPL2, we speculated that the putative SPL2 affinity for Ln ions might not be as high as in the case of designed LBTs.

We also analyzed another homologous E3 ligase found in the chloroplast outer membrane, the SPL1 protein. Not surprisingly, both RING domains of SPL1 and SPL2 are strikingly similar (Figure 1C). On the contrary, the linker region between the transmembrane domain (not shown) and the RING domain shares a very weak sequence homology. In the case of SPL2, the linker region creates a highly charged stretch that can be divided into two parts: the basic motif is flanked on the N-terminal side by the hydrophobic transmembrane domain, and on the C-terminal side by a cluster of acidic residues located in close proximity to the RING domain. In total, charged amino acid residues account for around 41% of this motif, of which 26% are acidic residues. In the case of SPL1, basic residues account for 27% of this region. In addition, they are not clustered as in the SPL2 protein but are dispersed across the linker sequence. Taken together, we hypothesize that the putative LBT is localized to the linker region of SPL2.

### 2.2. Emission Spectra of SPL2cyt and Protein–Tb^3+^ Complexes

Since the putative LBT is found in the cytosolic region of SPL2 from *A. thaliana*, for further work, we expressed and purified only the truncated form of SPL2, henceforth SPL2cyt. Notably, this fragment of the protein contains a total of three tryptophan residues located close to each other (W294, W297, W300) in the long α-helix of the SPL2cyt amino-terminal region. In fact, the unusual abundance of tryptophan in membrane proteins has been reported [13], where these residues are non-randomly preferred near the membrane–water interface [14,15]. Therefore, we reason that W294, W297, and W300 of SPL2 serve as membrane anchors, embedding the protein in chloroplasts of *Arabidopsis*.

**Figure 2:**
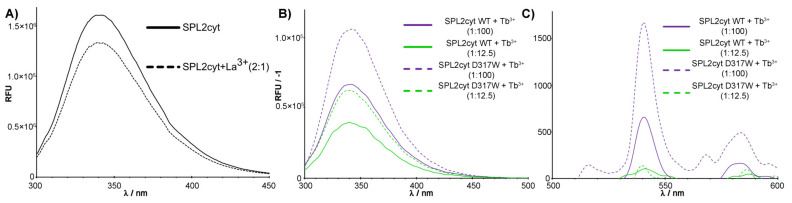
Fluorescence spectra in the presence of different Ln ions. (**A**). Tryptophan fluorescence spectra of 2 μM SPL2cyt in the absence (dotted line) and presence (solid line) of La^3+^ at indicated molar ratios. (**B**) Normalized tryptophan fluorescence spectra and (**C**) normalized terbium luminescence spectra of 2 μM SPL2cyt (WT) and its mutant (D317W) in the presence of Tb^3+^ at indicated molar ratios. Each spectrum was background-corrected for a protein-free buffer containing indicated concentrations of terbium ions.

The presence of three tryptophan residues in the SPL2 protein is easily detected by the emission fluorescence spectrum, which shows λ_max_ at 340 nm (Figure 2A). In agreement with our predicted structure, this emission spectrum is characteristic of solvent-exposed tryptophan residues. Additionally, the presence of these tryptophan residues in the SPL2cyt sequence and their emission properties can be used to probe inter-domain structural changes occurring upon the metal binding to the protein. Here, we show that in the presence of La^3+^, the tryptophan emission intensity decreases, and the emission spectrum undergoes a moderate shift to a lower wavelength of 339 nm. Similarly, the presence of another Ln ion, terbium, also resulted in decreased tryptophan emission of SPL2cyt (Figure 2B). Fluctuations in the intrinsic protein fluorescence could be due to Tb^3+^-induced conformational changes in the protein, such as tryptophan quenching by energy transfer to Tb^3+^.

To confirm our initial findings, we carried out further measurements of the Tb^3+^ emission intensity since they provide a qualitative means for observing Tb^3+^ binding to proteins due to excitation by energy transfer from tryptophan residues. Indeed, upon addition of Tb^3+^, characteristic emission in the visible region occurred (Figure 2C). To further evaluate this effect, we performed site-directed mutagenesis and chose D317 to be substituted with tryptophan. Among the canonical amino acids, tryptophan is the most effective luminescence donor, even though tryptophan is primarily limited to the efficient sensitization of Tb(III) and Eu(III). At the same time, we precluded the side chain of D317 as a metal-coordinating ligand on the basis of sequence alignments with iteratively designed LBTs and our predicted structure. The localization of D317 in the loop ensured that the tryptophan indole side chain projects to the solvent yet would still be within 10 Å of the bound metal, providing its sensitization. Indeed, the introduction of the additional tryptophan residue resulted in the most prominent luminescence attributed to Tb–protein complexes (Figure 2C).

**Figure A2:**
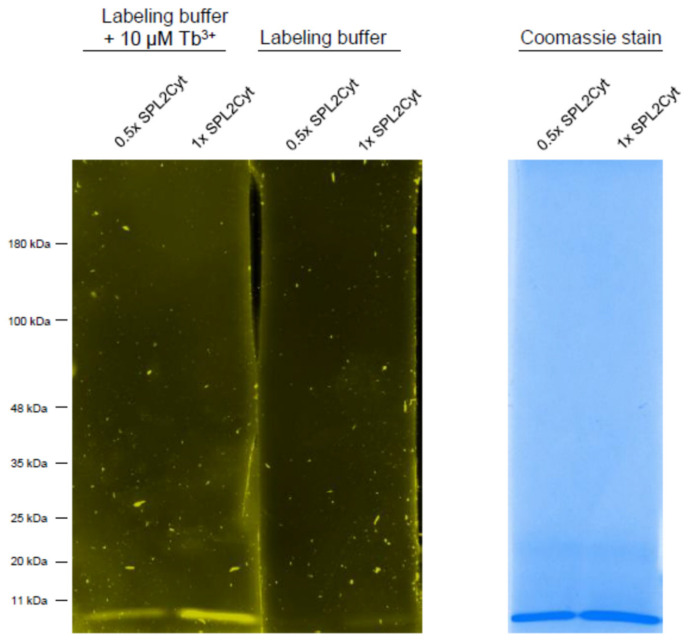
Visualization of the SPL2cyt protein after SDS-PAGE after incubation with Tb^3+^ or Coomassie stain.

The presence of Ln-binding sequences often enables rapid luminescent visualization on gels [16]. Indeed, upon soaking in the buffer supplemented with Tb^3+^, SPL2cyt was readily stained, thus being able to provide sufficient binding and brightness for convenient detection in sodium dodecyl sulfate (SDS) polyacrylamide gels (Figure A2, Appendix A). Therefore, we concluded that the SPL2cyt protein contains a lanthanide-binding motif.

### 2.3. CD Measurements and Metal Binding of SPL2cyt and Its Mutants

**Figure 3:**
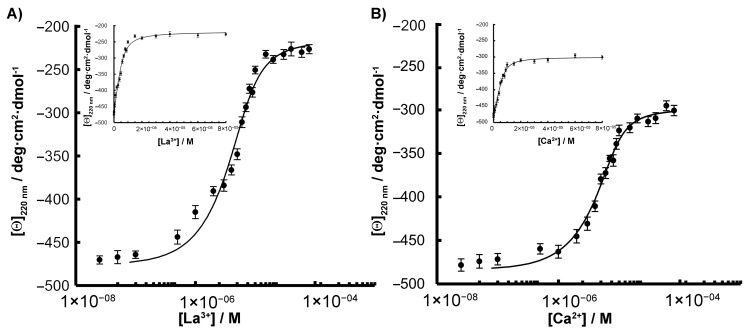
Metal titrations of 8 μM SPL2cyt with (**A**) La^3+^ and (**B**) Ca^2+^ monitored by CD spectroscopy. Data were fitted to the Hill equation to determine Kd and are reported in Table 1. Binding titrations plotted with a linear scale are also shown (insets).

Additional data confirming Ln binding by the SPL2cyt protein in vitro were obtained from studying the changes in the protein structure caused by its interaction with Ln ions. To perform the calculation of the protein–metal disassociation constants, *K*_d_, SPL2cyt was analyzed by circular dichroism (CD). The results are summarized in Table 1, and a typical titration curve is shown in Figure 3A. Many LBTs that are composed of natural amino acids and harboring aspartic acid residues as metal-coordinating residues show dissociation constants in the micromolar range [12,17]. This corresponds very well with the value reported here for SPL2cyt (*K_d_* = 0.95 μM). When the obtained *K*_d_ values were compared with *K*_d_ of LanM for La^3+^ ions, which is on the order of 10^−12^ M [18], the lanthanide-binding site on SPL2cyt can be regarded as a medium-affinity site. However, LanM represents a unique protein in terms of both affinity and selectivity towards Ln ions. Therefore, we conclude that SPL2cyt should be compared with other LBTs, such as the above-mentioned lanthanide finger proteins.

**Figure A3:**
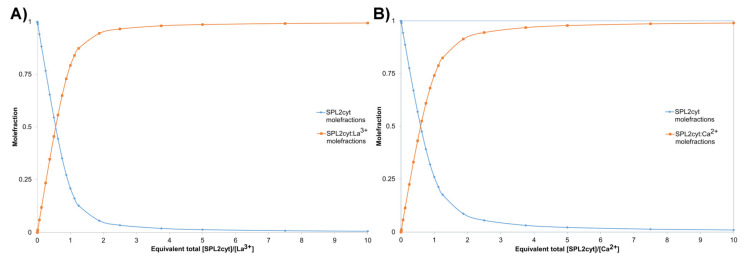
The stoichiometry of binding between the SPL2cyt protein and (**A**) La^3+^ and (**B**) Ca^2+^. Data were fitted using supramolecular.org [51].

In the next step, we determined the binding constants of calcium ions as many Ca^2+^-binding sites are also known to bind Ln^3+^ due to remarkable similarity in their sizes, bonding, and coordination geometry [19]. The similarity of trivalent lanthanides to divalent calcium also explains why early artificial lanthanide-binding sequences were derived from native calcium-binding proteins [20]. In the case of Ca^2+^ binding to the SPL2cyt protein, experimental data fitted to the Hill equation yielded the dissociation constant value of 1.21 μM. The obtained *K*_d_ value is around 90 times lower than the typical value observed for high-affinity Ca^2+^-binding proteins such as parvalbumin [21], but ca. 60 times higher than values reported for other Ca^2+^-binding proteins such as PsbO [22]. In both cases of Ca^2+^ and La^3+^, metal–protein binding stoichiometry was determined as 1:1 (Figure A3, Appendix A).

**Figure 4:**
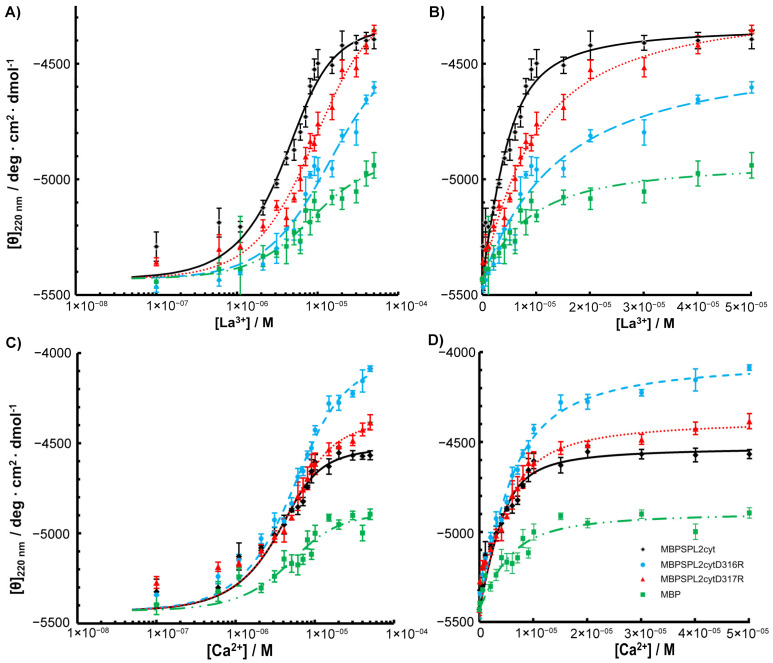
(**A**) La^3+^ and (**C**) Ca^2+^ titrations of MBP-SPL2cyt (black) and its mutants (MBP-SPL2cytD316R (blue), MBP-SPL2cytD317R (red)) measured by spectrapolarimetry. (**B**,**D**) Binding titrations of La^3+^ and Ca^2+^, respectively, plotted with a linear scale.

**Figure 5:**
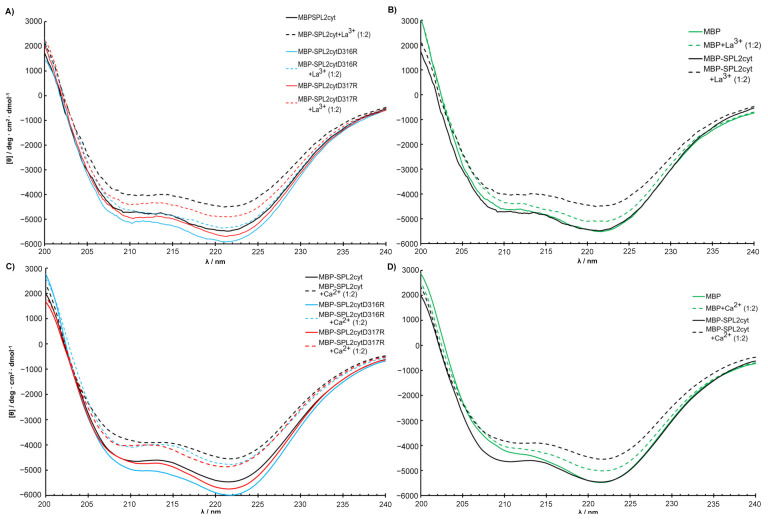
Structural changes in MBP-SPL2cyt and its mutants upon binding of (**A**) La^3+^ and (**C**) Ca^2+^ measured by CD. (**B**,**D**) Effects of La^3+^ and Ca^2+^, respectively, on the conformation of MBP and MBP-SPL2cyt. For direct comparison of MBP and MBP-SPL2cyt, spectra shown in (**B**,**D**) were normalized at 220 nm.

To assess the effects of amino acid substitutions on the binding of La^3+^, we created SPL2 mutants where D316 or D317 were substituted with arginine residues (D316R and D317R, respectively). Unfortunately, we were unable to obtain soluble SPL2-D316R upon removal of maltose-binding protein (MBP), a protein tag that enhances solubility, which we use for recombinant protein expression. Therefore, a part of the experimental work was performed employing a protein fused to MBP. DLS measurements revealed that both mutations resulted in increased hydrodynamic radii with respect to the wild-type protein (Table 1). This may suggest protein aggregation; however, upon removal of the aggregated fraction, MBP-SPL2cyt-D316R is still able to bind La^3+^ (Figure 4) at ca. one order of magnitude lower affinity than MBP-SPL2cyt (11.3 μM vs. 1.72 μM, respectively). In contrast, the dissociation constant of the second mutant, MBP-SPL2-D317R, is more similar to that of MBP-SPL2cyt (6.8 μM vs. 1.72 μM, respectively). These observed differences may result from the fact that the aspartic acid at position 316 is conserved among aligned sequences (Figure 1) and has been confirmed to coordinate metal ions in other LBTs. As a control, we also measured *K*_d_ of MBP-SPL2cyt, which falls within the range of the MBP-free SPL2cyt (1.72 μM vs. 0.95 μM, respectively), providing evidence that MBP itself does not significantly affect the binding of lanthanide ions. In addition, both mutations exerted a minor effect on the binding of Ca^2+^ (Figure 4 and Table 1) and the protein secondary structure (Figure 5).

### 2.4. Interactions with Ca^2+^ and La^3+^ Induce Structural Transitions in SPL2cyt

**Figure 6:**
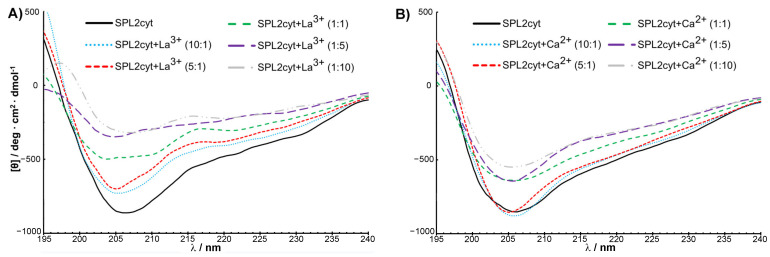
SPL2cyt undergoes a conformational change in the presence of La^3+^ or Ca^2+^ ions. Far-UV CD spectra of SPL2cyt (8 μM) in the presence of (**A**) La^3+^ or (**B**) Ca^2+^ at indicated molar ratios.

To gain first insights into structural features of the SPL2cyt protein, circular dichroism (CD) spectra of the recombinant protein in the far-UV (195–240 nm) region were measured. Consistent with the overall similarity of RING finger protein sequences, the secondary structure of SPL2cyt in its native state represents a mixture of α-helices, β-sheets, β-turns, and unordered regions, with the domination of α-helices. Remarkably, the SPL2cyt possesses some features characteristic of intrinsically disordered polypeptides, as evidenced by the strong negative peak at 206 nm (Figure 6). This observation is also in good agreement with our in silico analysis and with known structures of E3 ligases where the RING domain constitutes the structured region composed of aforementioned secondary structure motifs, while the remaining parts are often described as disordered regions [23].

**Figure A4:**
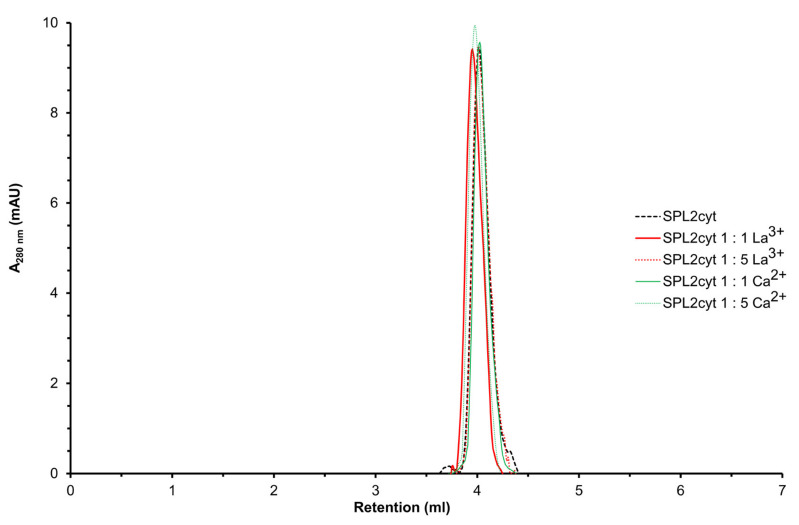
Analytical size-exclusion chromatography of SPL2cyt in the absence or presence of La^3+^ or Ca^2+^ at indicated molar ratios.

Encouraged by preliminary luminescence data that provided the first evidence for interactions between Tb^3+^ and SPL2cyt, we investigated metal binding by complementary techniques such as CD. The application of CD for monitoring the changes in the secondary structure of a protein upon interaction with ligands is well documented. Following the addition of La^3+^, a 1 nm blueshift of the negative peak is detectable, indicating that SPL2cyt undergoes a conformational change (Figure 6A) which is, however, not detectable by the SPL2cyt elution profile from size-exclusion chromatography (Figure A4, Appendix A). More importantly, the CD spectrum of SPL2cyt shows a significant increase in ellipticity at 205 and 220 nm corresponding to a loss of α-helical and β-sheet structure in the protein. This change in the CD spectrum was observed even at low concentrations of La^3+^ and may implicate a transition to a random coil composition upon the metal binding. Interestingly, low concentrations of Ca^2+^ did not induce such prominent changes in the UV region of the CD spectrum; however, the conformation of SPL2cyt was still affected but at higher Ca^2+^ concentrations probed here. Nevertheless, this effect is not as drastic as changes induced by La^3+^ ions (Figure 6B). The spectra recorded in the presence of La^3+^ or Ca^2+^ binding show that both ions affect the protein secondary structure, and differences between spectra could arise from the different binding constants of Ca^2+^ and La^3+^ (Table 1).

**Figure 7:**
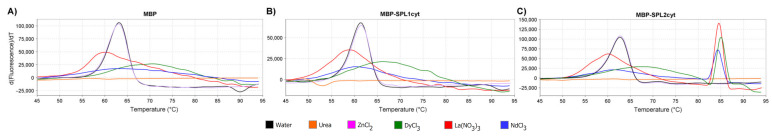
First derivatives of the melt curves of (**A**) MBP, (**B**) MBP-SPL1cyt, and (**C**) MBP-SPL2cyt. All proteins were assayed at 10 μM in the presence of indicated salts (2 mM) or urea at 4 M final concentration.

Apart from structural transitions, the interaction between ligands and proteins may induce changes in protein thermal stability. We investigated this possibility by measuring changes in the thermal denaturation point using differential scanning fluorimetry. Zinc ions, which are natively present in the RING domain of E3 ligases, did not affect the protein stability, unlike lanthanide ions, which, in turn, significantly increased the thermal stability of MBP-SPL2cyt (Figure 7C). Importantly, neither MBP nor MBP-SPL1cyt showed any signs of stabilization in the presence of the assayed metal ions (Figure 7A,B, respectively).

### 2.5. SPL2 Auto-Ubiquitination in the Presence of Lanthanide Ions

**Figure 8:**
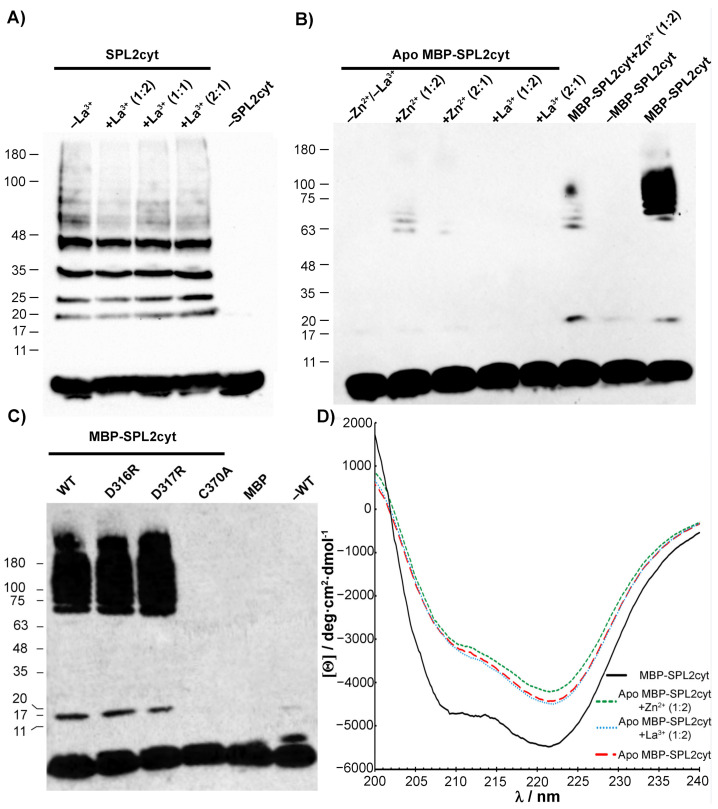
Analysis of SPL2cyt and MBP-SPL2cyt auto-ubiquitination activity in vitro. (**A**) Enzymatic assay of SPL2cyt in the absence or presence of La^3+^ at indicated molar ratios. (**B**) Activity of zinc-free (apo) MBP-SPL2cyt after incubation with Zn or La ions. For comparison of enzymatic activity, holo MBP-SPL2cyt is also shown. (**C**) Effects of indicated mutations in the putative LBT region of MBP-SPL2cyt (D316R and D317R) and RING (C370A) on auto-ubiquitination activity. Wild-type MBP-SPL2cyt protein (WT) is shown for comparison. All Western blots were analyzed with anti-ubiquitin antibodies to detect free ubiquitin and polyubiquitination products. (**D**) CD spectra and structural changes in SPL2cyt upon removal of Zn^2+^ and subsequent incubation with either Zn or La ions.

The activity of E3 ubiquitin ligases such as SPL2 can be assessed by their auto-ubiquitination ability, where specific residues—in most cases lysines—are modified. In the presence of E1, E2, ATP, and free ubiquitin, natively folded SPL2 is able to generate polyubiquitin chains that are covalently attached to SPL2. Since SPL2 is a metalloprotein belonging to the RING finger domain family, its activity relies on a proper folding of the zinc-binding motif. Indeed, we observed a distinct polyubiquitination pattern when we tested for the self-ubiquitination activity of SPL2cyt, confirming that the protein is folded correctly and functional. In the next step, we included La^3+^ ions in the reaction buffer to rule out the possibility that these ions could interfere with SPL2 enzymatic function. Importantly, neither of the molar ratios tested did affect the polyubiquitination pattern in a detectable fashion (Figure 8A) even though the presence of La^3+^ induces conformational changes in the native protein (Figure 6). Therefore, we conclude that La^3+^ ions do not substitute bound zinc ions in the RING domain. Instead, La^3+^ association with the LBT of SPL2 (Figure 1) promotes structural changes observed in the CD spectrum upon metal binding that do not impact the enzymatic activity.

To better elucidate the effect of Ln ions on auto-ubiquitination or the lack of thereof, we incubated SPL2 with EDTA. Removal of zinc ions by EDTA resulted in significant aggregation of apo SPL2cyt (Table 1), and any further measurements of its enzymatic activity were possible only in the case of SPL2cyt fused with MBP. As expected, the chelation of Zn ions by EDTA led to the loss of any detectable MBP-SPL2cyt enzymatic activity (Figure 8B). A very similar effect was observed when the critical C370 residue of the SPL2cyt RING domain was substituted by alanine to abolish the coordination of the Zn ion (Figure 8C). In contrast, neither mutation in the putative LBT affected the polyubiquitination pattern as both mutant proteins displayed robust ubiquitination, indicated by high-molecular-weight bands of varying size (Figure 8C). In the case of the apo form of MBP-SPL2cyt, its activity could only be partially recovered by the incubation of the unfolded protein with Zn^2+^. On the other hand, the incubation of the apoprotein with La^3+^ does not lead to the recovery of the auto-ubiquitination activity (Figure 8C).

In light of this, we investigated the effect of EDTA-induced Zn^2+^ removal on the secondary structure of MBP-SPL2cyt by CD spectropolarimetry. Following the addition of EDTA, MBP-SPL2cyt loses α-helical and β-sheet structures, as shown by drastic changes in ellipticity (Figure 8D). Even though the loss of Zn^2+^ does not result in the complete unfolding of the protein, it is accompanied by the loss of auto-ubiquitination activity (Figure 8B). This residual secondary structure, which is independent of Zn^2+^ coordination, could arise from fragments of the protein that fold in a zinc-independent manner, such as MBP, accounting for ~80% of the fusion protein. Strikingly, the presence of La^3+^ in the reaction mixture does not induce any detectable changes to the protein in the apo form, as evident by their overlapping CD spectra (Figure 8D). On the other hand, protein conformation changes moderately upon the addition of Zn^2+^, leading to the partial recovery of the enzymatic activity. In summary, the unfolding of the SPL2cyt RING domain is at least partially reversible, provided that the pool of Zn and not La ions is accessible.

## 3. Discussion

To date, various lanthanide ions have been reported in plant studies. For instance, Dy^3+^ and Eu^3+^ were used to probe Ca^2+^-binding sites in photosystem II [22,24], and Gd^3+^ was applied to study calcium channels in the ER of higher plants [25]. In addition, several reports exist that show that many non-related enzymes retain their abilities upon binding of Ln ions. This includes very well-studied Ca-binding proteins such as calmodulin from pea seedlings [26] and α-type phospholipase D from white cabbage [27], as well as tobacco Mg-binding proteins, such as ribulose bi-phosphate carboxylase [28] and Mg-ATPase [29]. To the best of our knowledge, we report here for the first time an example of a new class of enzymes, an E3 ligase that binds some Ln ions, i.e., La^3+^ and Tb^3+^.

E3 ubiquitin ligases catalyze the covalent attachment of a small protein modifier, ubiquitin, to many substrates in eukaryotic cells. The Really Interesting New Gene (RING) represents a large type of these enzymes that are widespread across the plant kingdom [30], where they are often important for growth and adaptation to stressful conditions. In *Arabidopsis*, the ubiquitin E3 ligase that localizes to the chloroplast’s outer membrane, the SP1 protein, promotes plant responses to several abiotic stress conditions, such as osmotic, oxidative, and salt stresses [31]. Since abiotic stresses account for a major cause of agricultural yield losses [32], long-term studies of the SP1 protein and its homologs may result in obtaining plants with improved yield-related traits.

Although several reports focused on SP1 have shown its important roles in plant development and various stress responses, detailed studies of SPL1 and SPL2 are not available, and thus their roles remain elusive. In the case of SPL2, we were able to identify a region encoding a putative lanthanide-binding tag (LBT), a striking feature that distinguishes this E3 ligase from the other two types of E3 ligases embedded in the chloroplast outer membrane, SP1 and SPL1. More specifically, this putative LBT is located in the vicinity of the chloroplast outer membrane, in the linker region between the second transmembrane domain and RING, and thus is exposed to the cytosol. Titrations of the cytosolic fragment of SPL2, SPL2cyt, with La as well as Ca ions resulted in the weakening of the negative ellipticity, which is an indication of the binding and the formation of a looser protein conformation. These observations indicate that the binding of all investigated ions to the protein causes similar structural changes. However, we noted some concentration-dependent differences between La and Ca ions. While even low concentrations of La^3+^ affected the protein secondary structure, the higher concentration of Ca^2+^ was required to exert similar effects. Despite overall similarities between these ions, we do not rule out a possibility that more subtle structural changes occur upon binding of different metal ions, but these are not visible in the recorded CD spectra.

A comparison of available RING domain structures shows that all RING fingers adopt a similar fold with the so-called “cross-brace” motif for zinc ligation. Its overall structure is characterized by the βαβ motif that unfolds in the presence of zinc chelators. Similarly to other RING fingers, both α-helices and β-sheets are detectable in the CD spectra of SPL2cyt, and the protein undergoes a structural rearrangement upon the addition of EDTA. While the protein unfolding results in the lack of SPL2 auto-ubiquitination, changes in the protein secondary structure that are induced by Ln ions do not affect the polyubiquitination pattern of SPL2. This indicates that the binding of Ln ions could be biologically compatible, as the protein retains its enzymatic functionality.

Even though the physiological significance of Ln binding to SPL2, if any, is not known, our results still represent an interesting finding as one can envision the possibility of labeling SPL2 with Ln ions to identify its interaction partners. This often requires putative substrates to be fused with fluorescent proteins. In many cases, techniques such as the bimolecular fluorescence complementation (BiFC) assays have been routinely used to study protein–protein interactions and their subcellular localization in plants, especially since several advances have been made in the field [33,34]. Similarly, Förster resonance energy transfer (FRET) with fluorescent proteins is also applied for imaging of protein–protein interactions in living cells. Unfortunately, both BiFC and FRET suffer from inherent difficulties, such as background fluorescence, which can obscure weak signals. This major problem can be addressed by lanthanide-based resonance energy transfer (LRET), where a time-gate delay of 10–100 μs is employed between probe excitation and signal acquisition to eliminate ns-long background fluorescence signal. Indeed, rapid development of this emerging method has been observed in recent years, as luminescent complexes of lanthanide cations (Tb^3+^ and Eu^3+^, in particular) have been employed as donor chromophores to fluorescent proteins [35,36,37]. In light of this, the binding of Tb^3+^ could be, in principle, employed to identify SPL2 ubiquitination substrates and E2s in vivo.

To better elucidate the relationship of the SPL2 protein to other E3 ligases belonging to the RING family, we performed sequence comparisons and revealed its presence across plant lineages. While this finding is not surprising, it is noteworthy that the SPL2 family is found (data not shown) in several agricultural important species (soybean, cotton, black tea, coffee), vegetables (cucumber, artichoke), fruits (grapes, pomegranate), drupes (almond, peach), and trees (oak, poplar). Even though lanthanides are widely regarded as biologically non-essential elements, low concentrations of at least some REEs result in improved plant growth and yields. A similar effect could also be observed for other photosynthetic organisms, such as the green alga *Desmodesmus quadricauda* [38], and the increase in the photosynthetic rate observed in the presence of Ln has been ascribed to the replacement of the central Mg ion of chlorophyll by REEs [25,26]. While increased crop yields already represent a reason to supplement soil with REEs [5], their role in the phytoremediation of lanthanides from soil and water also deserves attention [39].

**Figure 9:**
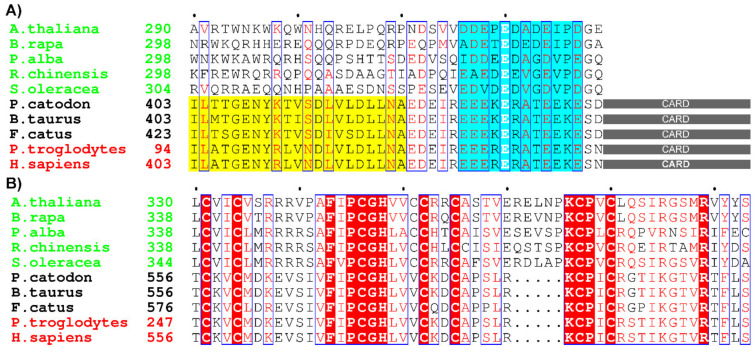
Selected sequences retrieved from BLAST searches of E3 ligases harboring LBT in plants (green), animals (black), and primates (red). (**A**) Lanthanide-binding motifs(cyan) present in some plant and animal E3 ligases. The ubiquitin-associated (UBA) domain of cIAP2 is highlighted in yellow. The caspase activation and recruitment domain (CARD) is shown as gray rectangles. (**B**) RING domains of selected E3 ligases. White letters on a red background represent strictly conserved residues; similar residues are shown as red letters on a white background in boxes.NCBI reference sequences are listed in Materials and Methods.

In addition, and interestingly, our preliminary results identified several other LBT-containing E3 ligases from the animal kingdom. Remarkably, their putative LBTs share very high sequence similarity with the motifs that we identified in plant E3 ligases (Figure 9A). Interestingly, all the identified proteins belong to the family of IAPs (inhibitor of apoptosis proteins), with the most prominent example of human BIRC3 (baculoviral IAP repeat containing 3; also known as cIAP2). This protein comprises the BIR3 domain followed by the ubiquitin-associated (UBA) domain, the caspase activation and recruitment domain (CARD) involved in autoinhibition of its E3 ligase activity, and the C-terminal RING domain with E3 ubiquitin ligase activity that mediates proteasomal degradation of cellular targets as well as itself [40] (Figure 9B). Clearly, a fluorescence approach or CD measurements will be needed to expand the scope of our findings to confirm the binding of Ln ions to cIAP2. Given that IAPs are regarded as therapeutic targets [41], their structures have been extensively studied. Unfortunately, all the reported constructs used for structural analysis of cIAP2 are limited to BIR and RING domains and thus are deprived of LBTs [42,43,44]. This indicates that novel crystallization or NMR studies are needed to gain insight into the interactions between Ln ions and these proteins.

## 4. Materials and Methods

### 4.1. Protein Expression and Purification

Wild-type and mutated variants of SPL2cyt were expressed in *Escherichia coli* strain BL21 (DE3) according to [45]. Protein expression was induced by 0.1 mM IPTG. After 3 h of expression, cells were harvested by centrifugation and the pellet was frozen and kept at −80 °C for further processing.

Cells suspended in 25 mM HEPES/KOH pH 8, 200 mM KCl, 50 mM arginine, 50 mM glutamate, 25 mM citrate, 0.1 mM ZnSO_4_, 2 mM TCEP containing Halt™ EDTA-free protease inhibitor cocktail (Thermofischer Scientific, Waltham, MA, USA) and OMNI nuclease (EurX, Gdansk, Poland) were passed through a Microfluidizer LM20 at 15,000 psi and centrifuged at 26,000× *g* for 30 min at 4 °C. Proteins were first passed through a 5 mL MBPTrap column (Cytiva, Chicago, IL, USA) and subsequently loaded onto a HiLoad 16/600 Superdex 200 pg column (Cytiva, Chicago, IL, USA).Only fractions containing monomeric fusion proteins were pooled, aliquoted, and stored at −80 °C.

For further details of cloning and protein purification, please refer to Appendix B.

### 4.2. Gel Electrophoresis, In-Gel Luminescence, and Immunoblotting

To assess protein purity and for the analysis of auto-ubiquitination assays, proteins were resolved using denaturing Tricine-PAGE [46]. In-gel luminescence was observed under native conditions using Tris-glycine gels [47]. After electrophoresis, the gel was washed in a buffer containing 10 mM HEPES/NaOH pH 7 and 100 mM NaCl for 10 min, and subsequently in the fresh buffer supplemented with 5 µM TbCl_3_ for 1 h at room temperature. Tb^3+^ luminescence was detected on a ChemiDoc Imaging System (Bio-Rad, Hercules, CA, USA) using 302 nm for excitation and a wide band-pass filter (520–640 nm) for emission. As a control, a gel incubated in a buffer without TbCl_3_ was visualized simultaneously. Relative densitometry analysis was performed using ImageLab software (Bio-Rad) with the wild-type SPL2cyt as reference.

Antibodies were as follows: anti-Human Ubiquitin antibody (clone 83406, Biotechne, Minneapolis, MN, USA), 1:5000 dilution in TBS supplemented with 1% non-fat milk; anti-Mouse IgG HRP-coupled secondary antibody (Cell Signaling Technology, Danvers, MA, USA) at 1:10,000 dilution in TBS supplemented with 0.5% non-fat milk.

### 4.3. In Vitro Ubiquitylation Assay

For all auto-ubiquitination assays, 2.5 µM bacterially-expressed proteins were incubated with 28 nM 6× HisUBE1 (Biotechne, Minneapolis, MN, USA), 400 nM 6× HisUBE2D4 (Biotechne, Minneapolis, MN, USA), and 50 µM 6× HisUbiquitin (Biotechne, Minneapolis, MN, USA) in a reaction mixture containing 40 mM Tris pH 7.8, 60 mM NaCl, 1 mM TCEP, and 10 mM ATP/MgCl_2_. Samples were incubated for 3 h at 26 °C and reactions were stopped by adding SDS-PAGE Loading Buffer A at a 3:1 ratio. For assessment of SPL2cyt’s auto-ubiquitination rate in the presence of La^3+^ or Ca^2+^, equal reaction volumes were recovered to a separate tube and stopped at indicated time-points. All assays were analyzed by immunoblotting using anti-Ub, as indicated above.

### 4.4. Fluorescence Measurements

Measurements were performed on a spectrofluorometer FS5 (Edinburgh Instruments, Livingston, UK) at room temperature in a 10 mm clear-sided quartz cuvette. Intramolecular FRET to the Tb^3+^ acceptor was induced through the excitation of 2 µM SPL2cyt intrinsic fluorescence at the 280 nm wavelength and visualized by collecting emission spectra in the 300–600 nm wavelength range. The bandwidth slits were set to 3 and 2 nm for excitation and emission, respectively, and a 1 nm data collection interval with a 10 nm∙s^−1^ scanning speed was applied. Three accumulations were collected for each measurement after 10 min equilibration for each Tb^3+^ concentration point. Spectra were background-corrected for a protein-free Buffer B containing the corresponding TbCl_3_ concentration. Since the SPL2D_317_W mutant contains an additional tryptophan residue, as manifested by a different baseline value preventing simultaneous graphic comparison, the spectra were normalized by subtracting the background-corrected spectra of a TbCl_3_-free Buffer B containing 2 µM protein.

### 4.5. Circular Dichroism (CD) Spectroscopy

Circular dichroism (CD) spectra were recorded using a J-1500 Jasco spectropolarimeter (JASCO, Tokyo, Japan) at 25 °C in a 1 mm (all MBP-SPL2cyt variants) or a 2 mm (SPL2cyt) quartz cuvette, under a constant nitrogen flow. The mean spectra were derived from five accumulations in the range of 195–240 nm and 200–240 nm for SPL2cyt and all MBPSPL2cyt variants, respectively. All spectra were collected at a 200 nm∙min^−1^ scanning speed, a 0.2 nm data pitch, and a 1 nm bandwidth. For each La^3+^, Ca^2+^,or Zn^2+^ concentration data-point, spectra were collected after the equilibrium was reached, typically after 5–10 min. Spectra were background-corrected for Buffer B containing the given metal concentration. Protein dilution resulting from the addition of metal solutions was accounted for and corrected for the calculation of the molar ellipticity. Metal solutions were prepared as 20 mM stocks in TCEP-free Buffer B and were diluted at desired concentrations prior to mixing with the protein solution.

Titration experiments were conducted as above, except that the equilibration times were typically much shorter (1–3 min) due to a smaller metal concentration change between steps. The molar ellipticity values at 220 nm were extracted from the spectra and plotted as a function of the total metal concentration. Data-points represent an average of three independent measurements, while error bars indicate standard deviation. Calculation of the protein–metal disassociation constants, K_d_, was achieved by fitting the data to Equation (1) [48]:

(1) $${\left[\Theta \right]}_{220}={\left[\Theta \right]}_{0}+\left({\left[\Theta \right]}_{\mathrm{Sat}}-{\left[\Theta \right]}_{0}\right)\frac{\left(\left[P_{t}\right]+\left[M_{t}\right]+K_{d}\right)\pm \sqrt{{\left(\left[P_{t}\right]+\left[M_{t}\right]+K_{d}\right)}^{2}-4\left[P_{t}\right]\left[M_{t}\right]}}{2\left[P_{t}\right]}$$

where [Θ]_220_ is the experimental molar ellipticity value at 220 nm, [Θ]_0_ is the protein’s initial [Θ]_220_, [Θ]_Sat_ is the [Θ]_220_ of a metal saturated protein, [P_t_] is the total protein concentration (assumed constant), and [M_t_] is the known total metal concentration. K_d_ and [Θ]_Sat_ values were derived from fitting.

### 4.6. Analytical Size-Exclusion Chromatography

Hydrodynamic properties of proteins were analyzed using a TSKgel SuperSW2000 4.6 × 300 mm column (Tosoh Biosence, Tokyo, Japan). Prior to separation, the column was equilibrated in Buffer B pH 7, and 10 µM protein solutions were injected. Runs were performed at room temperature at a flow rate of 0.3 mL min^−1^. Bovine ribonuclease A and aprotinin (Cytiva, Chicago, IL, USA) were used as calibration standards. All elution profiles were baseline-corrected and were not further normalized. In each case, the most representative elution profile of three repeated runs is shown.

### 4.7. Dynamic Light Scattering

Hydrodynamic diameter (D_h_) of analyzed proteins was assessed by DLS using a Zetasizer Nano ZS (Malvern Panalytical, Malvern, UK). Scattering of a 5 µM protein solution was measured in a low-volume 3 mm quartz cuvette (Malvern Panalytical, Malvern, UK) at 25 °C with refractive index and dynamic viscosity set as 1.33 and 0.8872 cP, respectively. Three separate measurements of 10–15 accumulations were averaged to obtain volume distribution-based D_h_. In the case of all measurements, peaks corresponding to obtained D_h_ values accounted for over 99% of sample scattering measured by volume.

### 4.8. Multiple Sequence Alignments and Structural Prediction

Sequences were aligned using the ClustalO web server [49] with default settings. NCBI reference sequence numbers of aligned sequences were as follows: NP_564653.1 (*A. thaliana*), XP_009147542.1 (*B. rapa*), XP_034900181.1 (*P. alba*), XP_024177586.1 (*R. chinensis*), XP_021865173.1 (*S. oleracea*), XP_007122198.1 (*P. catodon*), NP_001030370.1 (*B. taurus*), XP_019666764.1 (*F. catus*), XP_001151965.1 (*P. troglodytes*), (*H. sapiens*). Residue numbering and topology of SPL2 were derived from [8].

All the predicted 3D structural models were generated using the I-Tasser server [50].

### 4.9. Thermal Shift Assay

To assess the thermal denaturation point, proteins were assayed with the Durham Salt Screen (Molecular Dimensions, Ltd., Sheffield, UK). Final protein and SYPRO orange concentrations were 10 μM and 10×, respectively. Data were collected with a fluorescence reading being taken in every well at every temperature increment in a temperature gradient of 24–96 °C.

## 5. Conclusions

In summary, we have shown that SPL2 binds calcium and lanthanide ions. Even though metal binding induces structural rearrangements, the protein remains active, as evidenced by its unaffected auto-ubiquitination. Therefore, lanthanide-based resonance energy transfer could be used in the future to identify SPL2 upstream and downstream interacting protein partners.

## Figures and Tables

**Table 1:** Hydrodynamic diameter (Dh) in the presence or absence of La3+ and apparent dissociation constant (Kd) of SPL2 and its mutants.

Protein	D_h_ (nm)		*K_d_* (M)	
	−La^3+^	+La^3+^	+La^3+^	+Ca^2+^
SPL2cyt	n.d.	n.d.	9.45 × 10^−7^	1.21 × 10^−6^
MBP-SPL2cyt	7.72 ± 0.40	7.83 ± 0.27	1.72 × 10^−6^	1.13 × 10^−6^
MBP-SPL2cyt-D316R	8.99 ± 0.39	8.80 ± 0.22	1.13 × 10^−5^	3.03 × 10^−6^
MBP-SPL2cyt-D317R	9.19 ± 0.25	9.28 ± 0.20	6.80 × 10^−6^	2.09 × 10^−6^

**Figure A1:**
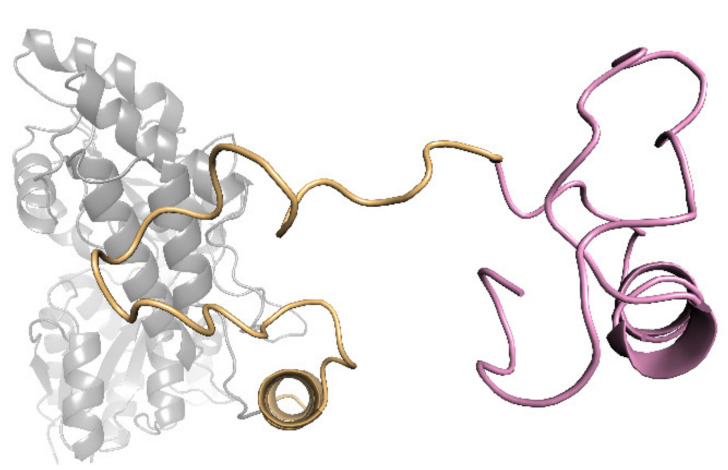
A modeled structure of the MBP-SPL2cyt protein. The orientation is as in Figure 1. MBP (gray), the linker region of SPL2cyt (gold), RING (pink).

## Data Availability

Not applicable.

## Appendix B

### Appendix B.1. Materials

All chemicals used in this study were purchased from renowned suppliers at the highest purity grade suitable for each application. All labware was flushed with the Milli-Q system (Merck, Darmstadt, Germany) type 1 ultrapure water before use. All cuvettes used in the study were incubated in a 2% Hellmanex III (Hellma, Zumikon, Switzerland) solution and washed with Milli-Q water between uses. All buffers were prepared using the Milli-Q water. Prior to use, Buffer B was incubated with the Chelex-100 resin overnight followed by a pH adjustment. Perfect Tricolor Protein Ladder (EurX, Gdańsk, Poland) was used as a protein molecular weight standard for electrophoresis and immunoblotting. ZnSO_4_, TbCl_3_, LaNO_3_, and CaCl_2_ were used as zinc, terbium, lanthanum, and calcium salts, respectively. Concentration of each metal stock solution was confirmed by a complexometric titration with a chelating agent (EDTA for Zn^2+^ or EGTA for Tb^3+^, La^3+^, and Ca^2+^) in the presence of xylenol orange as an indicator. Titrations were performed in a 20 mM MES/KOH pH 6.5 buffer.

### Appendix B.2. Cloning and Protein Expression

Codon-optimized genes encoding SPL2 (At1g54150) from *A. thaliana* were purchased from Life Technologies, Poland. The nucleotide sequence of the cytoplasmic domain (residues 291–383 [7]) was amplified by PCR and cloned into an N-terminal 6× HisTagMBP fusion protein pET-LIC expression vector containing a 10× Asn linker followed by a TEV protease cleavage motif. The *SPL2cyt* mutants, *SPL2D316R*, *SPL2D317R*, *SPL2D317W*, and *SPL2C370A* were generated by PCR-based site-directed mutagenesis of the expression vector. DNA sequences of all the resulting expression plasmids used in this study were confirmed by sequencing (Microsynth AG, Balgach, Switzerland).

Wild-type and mutated variants of SPL2cyt were expressed in *Escherichia coli* strain BL21 (DE3). Transformed cells were grown overnight in Miller’s LB medium containing 50 µg/mL of kanamycin at 37 °C with shaking. On the next day, 500 mL cultures were inoculated and grown to an OD_600_ of 0.2–0.3; the temperature was increased to 42 °C for 30 min, and the production of endogenous *E. coli* chaperones was stimulated by the addition of 0.1% glycerol and 0.1 mM potassium glutamate [45]. After heat shock, cells were grown at 37 °C with shaking to an OD_600_ of 0.6–0.7; when the temperature was lowered to 30 °C, the culture medium was supplemented with 0.1 mM ZnSO_4_ and protein expression was induced by 0.1 mM IPTG. After 3 h of expression, cells were harvested by centrifugation and the pellet was frozen and kept at −80 °C for further processing.

### Appendix B.3. Protein Purification, MBP-Tag Removal, Zinc-Free Protein Preparation, and Purity Assessment

The bacterial pellet containing the expressed protein was thawed and suspended in 50 mL of lysis buffer (25 mM HEPES/KOH pH 8, 200 mM KCl, 50 mM arginine, 50 mM glutamate, 25 mM citrate, 0.1 mM ZnSO_4_, 2 mM TCEP) containing Halt™ EDTA-free protease inhibitor cocktail (Thermofischer Scientific, Waltham, MA, USA) and OMNI nuclease (EurX, Gdańsk, Poland). Suspensions were passed through a Microfluidizer LM20 at 15,000 Psi and centrifuged at 26,000× *g* for 30 min at 4 °C. Supernatants were loaded on a 5 mL MBPTrap column (Cytiva, Chicago, IL, USA) equilibrated with Buffer A (25 mM HEPES/KOH pH 8, 200 mM KCl, 50 mM arginine, 50 mM glutamate, 2.5 mM citrate, 0.01 mM ZnSO_4_, 1 mM TCEP). Proteins were eluted with Buffer A supplemented with 30 mM maltose. The eluate was then loaded on a HiLoad 16/600 Superdex 200 pg column (Cytiva, Chicago, IL, USA) equilibrated with Buffer A. Fractions containing monomeric fusion protein were pooled, aliquoted, and frozen for storage at −80 °C after addition of 10% glycerol. No loss in stability and/or activity was observed upon thawing of all MBP-fused SPL2cyt.

To remove the N-terminal MBP tag, 27 nmol (1.5 mg) of purified fusion protein was treated with 100 U of TEV protease (EurX, Gdańsk, Poland) in a 1 mL reaction for 2 h at 26 °C. Digested sample was filtered through 0.2 µm PES and loaded on a HiLoad 16/600 Superdex 75 pg column (Cytiva Chicago, IL, USA) equilibrated with Buffer A. Fractions containing monomeric SPL2cyt were collected, pooled, and concentrated on a 3000 MWCO Amicon filter (Merck Millipore, Burlington, MA, USA) up to 20 µM (0.226 mg/mL). Wild-type and mutated SPL2cyt (except the C_370_A mutant) remained stable in Buffer A for at least 48 h when stored at 4 °C.

For experimental purposes, proteins were always transferred to Buffer B (10 mM Tris/H_2_SO_4_ pH 8, 100 mM NaCl, 0.4 mM TCEP or 10 mM Tris/H_2_SO_4_ pH 8, 100 mM NaF in case of CD studies) using a 5 mL HiTrap desalting column. This procedure caused an abrupt loss of stability for the SPL2cytD_316_R mutant, while its MBP-fused counterpart, as well as all the remaining variants of SPL2cyt, remained stable in Buffer B for at least 24 h (72 h for MBP fusions) stored at 4 °C at concentrations of up to 20 µM.

To generate Zn^2+^-free SPL2cyt or MBP-SPL2cyt, 10 µM protein in Buffer B was incubated overnight in the presence of 25 mM EDTA at 4 °C. Next, the sample was dialyzed overnight against an EDTA-free Buffer B in 4 °C with one buffer exchange after 3 h. Protein was then recovered from the dialysis tubing and centrifuged for 10 min at 20,000× *g* at 4 °C and the supernatant was collected. Zinc removal caused precipitation of SPL2cyt; however, MBP-SPL2cyt remained in the solution, forming soluble aggregates.

Protein purity was assessed by SDS-PAGE gel stained with Coomassie blue G-250 dye. Protein concentration was determined from the UV protein spectra collected in a 10 mm quartz cuvette on a UV–Vis DU800 spectrophotometer, room temperature (Beckman Coulter, Brea, CA, USA). The mean absorbance value at the 280 nm wavelength of three scans and the sequence-derived molar extinction coefficient were incorporated into the Lambert–Beer equation to obtain the molar concentration. Molar extinction coefficients were as follows: 19,480 M^−1^ cm^−1^, and 24,980 M^−1^ cm^−1^ for all SPL2cyt variants and SPL2cytD_317_W, respectively; 87,320 M^−1^ cm^−1^, and 92,820 M^−1^ cm^−1^ for all MBPSPL2cyt variants and MBPSPL2cytD_317_W, respectively.
